# Emotional Processing of Personally Familiar Faces in the Vegetative State

**DOI:** 10.1371/journal.pone.0074711

**Published:** 2013-09-25

**Authors:** Haggai Sharon, Yotam Pasternak, Eti Ben Simon, Michal Gruberger, Nir Giladi, Ben Zion Krimchanski, David Hassin, Talma Hendler

**Affiliations:** 1 Functional Brain Center, Wohl Institute for Advanced Imaging, Sourasky Medical Center, Tel Aviv, Israel; 2 Department of Internal Medicine, Sourasky Medical Center, Tel Aviv, Israel; 3 Sackler School of Medicine, Tel Aviv University, Tel Aviv, Israel; 4 School of Psychological Sciences, Tel Aviv University, Tel Aviv, Israel; 5 Department of Neurology, Sourasky Medical Center, Tel Aviv, Israel; 6 Sagol School of Neuroscience, Tel Aviv University, Tel Aviv, Israel; 7 Rehabilitation Intensive Care Unit, Loewenstein Rehabilitation Hospital, Raanana, Israel; University of Montreal, Canada

## Abstract

**Background:**

The Vegetative State (VS) is a severe disorder of consciousness in which patients are awake but display no signs of awareness. Yet, recent functional magnetic resonance imaging (fMRI) studies have demonstrated evidence for covert awareness in VS patients by recording specific brain activations during a cognitive task. However, the possible existence of incommunicable subjective emotional experiences in VS patients remains largely unexplored. This study aimed to probe the question of whether VS patients retain a brain ability to selectively process external stimuli according to their emotional value and look for evidence of covert emotional awareness in patients.

**Methods and Findings:**

In order to explore these questions we employed the emotive impact of observing personally familiar faces, known to provoke specific perceptual as well as emotional brain activations. Four VS patients and thirteen healthy controls first underwent an fMRI scan while viewing pictures of non-familiar faces, personally familiar faces and pictures of themselves. In a subsequent imagery task participants were asked to actively imagine one of their parent's faces. Analyses focused on face and familiarity selective regional brain activations and inter-regional functional connectivity. Similar to controls, all patients displayed face selective brain responses with further limbic and cortical activations elicited by familiar faces. In patients as well as controls, Connectivity was observed between emotional, visual and face specific areas, suggesting aware emotional perception. This connectivity was strongest in the two patients who later recovered. Notably, these two patients also displayed selective amygdala activation during familiar face imagery, with one further exhibiting face selective activations, indistinguishable from healthy controls.

**Conclusions:**

Taken together, these results show that selective emotional processing can be elicited in VS patients both by external emotionally salient stimuli and by internal cognitive processes, suggesting the ability for covert emotional awareness of self and the environment in VS patients.

## Introduction

The Vegetative State (VS) is a severe disorder of consciousness defined as preserved wakefulness but with lack of any behavioral evidence of awareness of either self or environment [1]. However, recent fMRI studies have documented elaborate brain activations in VS patients when asked to perform a cognitive task [2], [3], presenting a strong case for covert awareness. Yet, the quality of this covert awareness cannot be adequately evaluated without addressing the question of whether these cognitive processes also elicit a subjective emotional experience. Emotion is a key component of our subjective experiencing of the world, including our sense of self [4], serving as an ever-present basic constitute of the quality of human consciousness. To gain a better insight into “what it feels like to be in the vegetative state” it is thus pertinent to examine these patients' ability to process not only cognition, but also emotion.

Moreover, the question of emotional awareness is relevant even in the absence of complex cognition, since mental existence cannot be equated solely with advanced interactive cognitive capabilities. This is especially so in the context of a severe brain disorder, considering that in other neurological and psychiatric disorders, causing cognitive impairment, even communicating patients fail to perform specific commands while still being aware and emotionally responsive to the environment [5]. The same is also true of healthy young children who are able to employ emotional processes to interpret and communicate with the environment well before developing highly structured cognition [6]. Accordingly, VS patients may conceivably still retain the capacity for emotional processing even in the absence of complex cognition, preventing them from fully complying with complex cognitive tasks while still having a subjective experience of the environment.

To date, only one case-report and two fMRI studies have applied a simple emotional stimulus in VS patients, by using the sound of the patient's own name spoken in a familiar voice. These studies documented isolated cortical activations in a few VS patients, but lack of control stimuli of unfamiliar voices and the fact that brain processing of one's own name may be entirely automatic [7] critically limit the interpretation of such findings. Interestingly, a recent study has demonstrated that the sound of other people's pain cries can activate the so called “pain matrix” in VS patients, including areas thought to relate to the emotional aspects of pain [8].

To further explore emotional awareness in VS patients, we employed the emotive effect of processing personally familiar faces which involves not only category-specific face perception but also cognitive capacities such as autobiographical memories [9], [10] and emotional processing of familiarity cues. Accordingly, we expected a hierarchical effect, characterized by a common response of the face specific area (i.e. the fusiform face area, FFA [9]) with additional selective responses to familiar faces in brain regions essential for emotional and self-awareness processing such as the amygdala and the anterior insula [10], respectively.

To better examine the crucial question of emotional awareness in VS patients we also applied the well described active-paradigm approach [11], which can provide a stronger case for covert awareness in nonresponsive patients. However, unlike purely cognitive based paradigms used thus far, we wished to explore the ability of VS patients to perform voluntary top-down brain modulation of emotional information. To this end we applied a guided imagery task in which participants were instructed to imagine one of their parent's faces. It was expected that, in addition to the face specific activation elicited by this imagery [12], the imagery of a parent's face would also elicit emotional reactivity in major limbic areas such as the amygdala.

## Methods

### Participants

Four patients and thirteen healthy controls participated in the study. Control subjects had no history of neurologic or psychiatric illnesses (N = 13, 8 females, age 29±9). All patients complied with the American Academy of Neurology [13] as well as the Coma Recovery Scale-Revised (CRS-R) [14] definitions of VS, and were all healthy prior to the initial event. Table 1 depicts the behavioral and clinical characteristics of all patients. Though patients' characteristics vary significantly (as is commonplace in studies of VS patients), including patients that are more likely to improve than others (e.g. recent traumatic brain injury vs. anoxic brain injury) presents an opportunity to better identify differences between patients in an attempt to pinpoint prognostic markers. All patients were assessed in the weeks following the scan on a daily basis in clinical wards (either neurological wards or specially designated neuro-rehabilitation wards) by physicians certified in neurology or rehabilitation. All patients underwent bedside examination and scoring at the day of the fMRI, before and immediately following the scan. In the period following the scan patients returned to their wars to continue evaluation on a daily basis as detailed for the time preceding the scan. Written informed consent approved by the ethics committee of Tel Aviv Sourasky Medical Center was obtained from all controls and legal guardians of the patients. As shown in Table 1, two patients later improved to a Minimally Conscious State (MCS): Patient 1 was diagnosed as MCS 3 months following the scan after displaying sustained visual pursuit and fluctuating response to simple motor commands. Patient 3 was diagnosed as MCS 2 months following the fMRI scan after displaying sustained visual pursuit and fluctuating simple goal-directed behavior. She was later diagnosed as emerging MCS after regaining simple verbal communication skills in the form of reproducible yes/no answers and sustained simple goal directed behavior.

**Table 1:** Clinical and Demographic Patient Data.

Patient	Gender	Age	Time from the initial event	Cause	Structural MRI At the day of the fMRI scan	CRS-R	Highest level behavior	Outcome at 1 year
**1**	Female	23	3 Months	Traumatic Brain Injury	Multiple small intra-cerebral hemorrhages, Bilateral hypodense white matter changes	7	Spontaneous eye-opening; flexion withdrawal	MCS after 3 months
**2**	Male	61	5 Years	Anoxic Brain Damage	Severe brain atrophy, Ventriculomegaly, White matter depletion, Severe cerebellar atrophy	5	Spontaneous eye-opening	VS
**3**	Female	54	2 Months	Traumatic Brain Injury	Several small intra-cerebral hemorrhages, Small chronic right frontal subdural hematoma	6	Spontaneous eye-opening; Oral movement	MCS after 2 months
**4**	Male	24	4 Months	Anoxic Brain Damage	Significant brain atrophy, Widespread white matter changes, Ventriculomegaly, Cortical calcifications	5	Spontaneous eye-opening; Oral movement	VS

### MRI acquisition and preprocessing

MRI scans were acquired on a 3.0T MRI scanner (Signa EXCITE; GE Healthcare) with a standard eight-channel head coil using gradient echo-planar imaging sequence of functional T2*-weighted images (TR, 3000 ms; TE, 35 ms; flip angle, 90° FOV, 20×20 cm; matrix size, 64×64) with 50 axial 3 mm slices with zero space covering the entire brain. Functional data were preprocessed using Brain Voyager QX v.2 program (Brain Innovation B.V, Maastrich, Netherlands). Anatomical scans included 3D spoiled gradient echo (SPGR) sequence (FOV = 250, matrix = 256*256, slice thickness = 1 mm). Data analysis were performed using Brain Voyager QX v.2.2 program (Brain Innovation B.V, Maastrich, Netherlands) and included standard functional MRI preprocessing steps as described below. Extra care was applied to head movement correction given that VS patients tend to move their head spontaneously. Head motions were corrected using a 6 parameter, rigid-body method (3 rotations and 3 translation axis) with the first volume as reference. For motion detection linear interpolation was used, while for motion correction sinc interpolation was used. EPI time series with head movements exceeding 5 mm at any axis were discarded. Slice time correction, a 3 Hz high pass temporal filtering and 4 mm spatial smoothing were then performed for all EPI time series.

### Fmri analysis

Patient data was analyzed as single-subject studies while healthy control data was analyzed at a group levelusing RFX model with corrections for multiple comparisons. Accordingly, patient anatomical data was normalized for AC-PC plane, while controls were normalized to Talairach space [15].

*Whole brain analysis* was based on separate general linear models (GLMs) created for each paradigm (e.g. the passive viewing of faces and the active imagery task detailed below). Activations were deemed significant with a p value <0.01 for the healthy control group and p<0.05 for individual patients (both FDR corrected).

*Region of interest (ROI) analysis* was driven by our a-priori assumption regarding face selective and emotion processing regions. For the face selective activity we focused on the fusiform face area (FFA). Since patients' brains displayed significant pathological anatomical variations FFA locations were not determined only functionally but further validated by: A. the regional selectivity of the BOLD activation to the face condition (faces > patterns contrast) B. event-related time course plots showing selectivity to face stimuli C. connectivity analysis showing functional connectivity compatible with known FFA network (e.g. contra lateral FFA). For the familiarity effect we focused on the amygdala and anterior insula which were chosen anatomically for each participant. All ROIS were chosen at a corrected p value of 0.01. Average time courses from the chosen ROIs were subsequently used in a functional connectivity (FC) analysis which examined the whole brain correlation with the seed ROI time course during a chosen condition compared to baseline. A threshold of p<0.001 for significant clusters, corrected at the whole brain level, was used to determine the brain areas that were significantly correlated with the seed ROI.

### fMRI paradigms

All subjects underwent a passive viewing task followed by an active imagery task while inside the fMRI scanner.

#### Passive face viewing

Viewing faces was done using a simple block design paradigm in which blocks of pictures of either faces or patterns were passively viewed. Faces included three levels of familiarity: unfamiliar faces, personally familiar (e.g. father, mother or close friend) and self. The familiar category was thus prepared individually for each subject with assistance from the subject's family. The pictures of the patients were all pictures dating from before the even causing the VS. All event pictures were at the same large-scale size of 24*18 cm, covering most of the screen. Each of the four condition blocks consisted of 9 pictures of faces or patterns, each presented for 900 ms with an inter stimulus interval of 100 ms. Blocks were interlaced with an inter trial interval that was 12 seconds long and consisted of a grey screen with a fixation point. This trial was repeated twice for each participant. A ‘Blank’ condition was added for 48 seconds before first stimulus and 30 seconds after last stimulus presentation. A 9 seconds long ‘don’t care' condition was added as the first stimuli block, made of painted houses and faces figures. Thus, the total length of the passive face viewing paradigm was 04∶15 minutes.

#### Active Face Imagery

In the active imagery task subjects were instructed to visualize one of their parents' faces. The paradigm consisted of two blocks with each block dedicated to a different parent. The beginning of each block consisted of 5 auditory instruction sentences (the duration of each was 3 seconds) followed by a quiet interval of 6 seconds, allowing the subject to perform the imagery task. Additional quiet intervals of 30 sec were added before the first and after the last stimulus. An auditory signal marked the end of the imagery block and a quiet interval of 15 seconds was placed between the two imagery blocks. 18 null seconds were added at the beginning of the test and dumped later in the analysis. Thus the total length of the imagery task was 03∶03 minutes.

## Results

### Task 1 -Passive viewing of familiar and unfamiliar face

**Figure 1:**
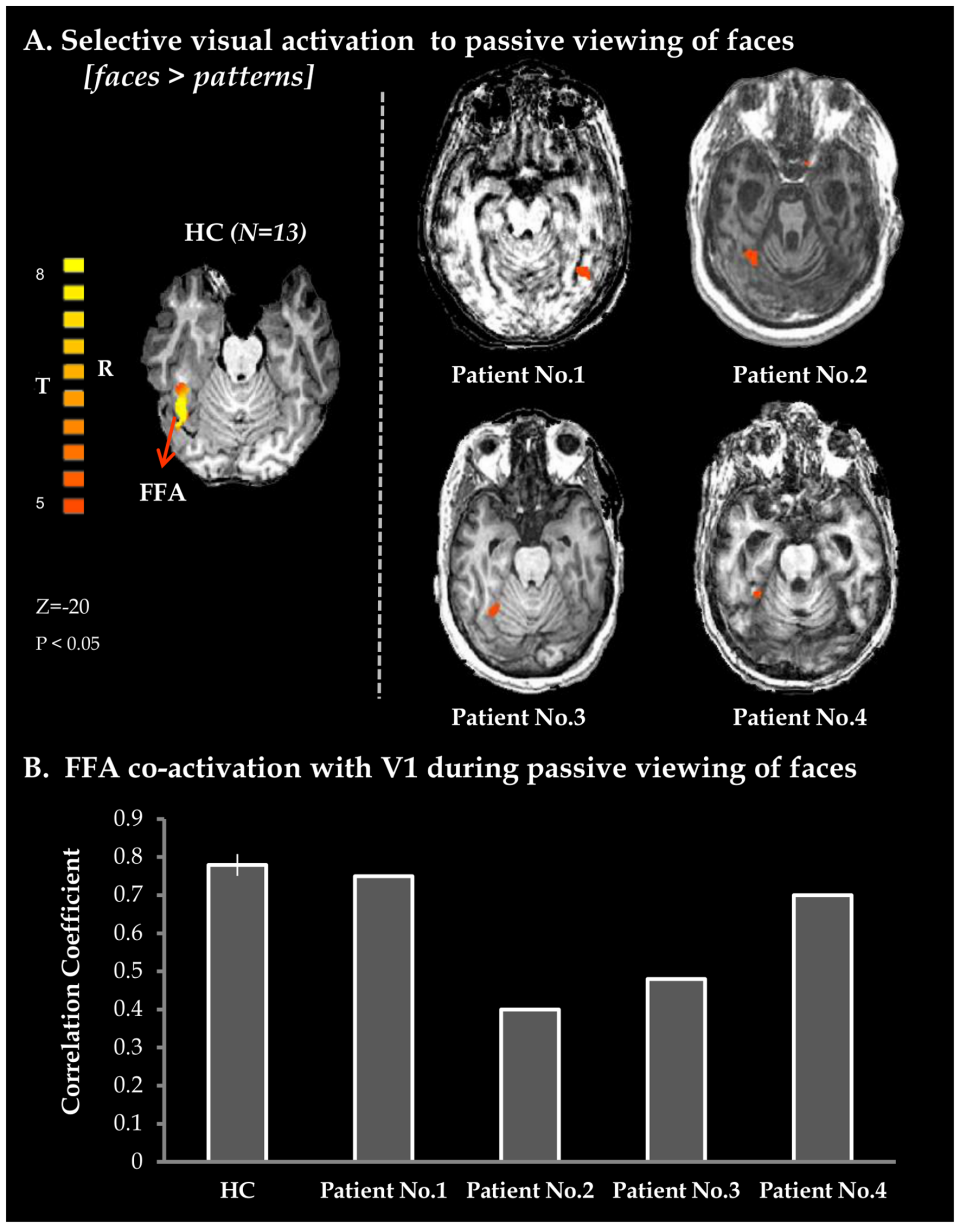
Perceptual processing of faces. A. Axial views of activation maps obtained from whole brain GLM analysis of Faces vs. Patterns in 13 healthy controls (extreme left, random effects FDR corrected (p<0.05)) and for individual VS patients (patient 1–4). BOLD activity maps for patients were superimposed on each individual's anatomical image. B. Average correlation-coefficient measures representing co-activation for FFA with V1 obtained from whole brain functional connectivity analysis with FFA as a seed region. Vertical black line denotes standard error in healthy controls (n = 13). HC – healthy controls; BOLD – blood-oxygenation-dependent level; FFA- fusiform face area; V1 – primary visual cortex.

As expected, viewing faces elicited robust activation in the Fusiform Face Area (FFA) in healthy controls as well as in all patients (see Figure 1A). We further found robust functional connectivity between the FFA and primary visual cortex (V1) in all patients, as in healthy controls (see Figure 1B). These activations provide the first consistent indication that VS patients are able to create specific visual brain representation of the environment.

**Figure 2:**
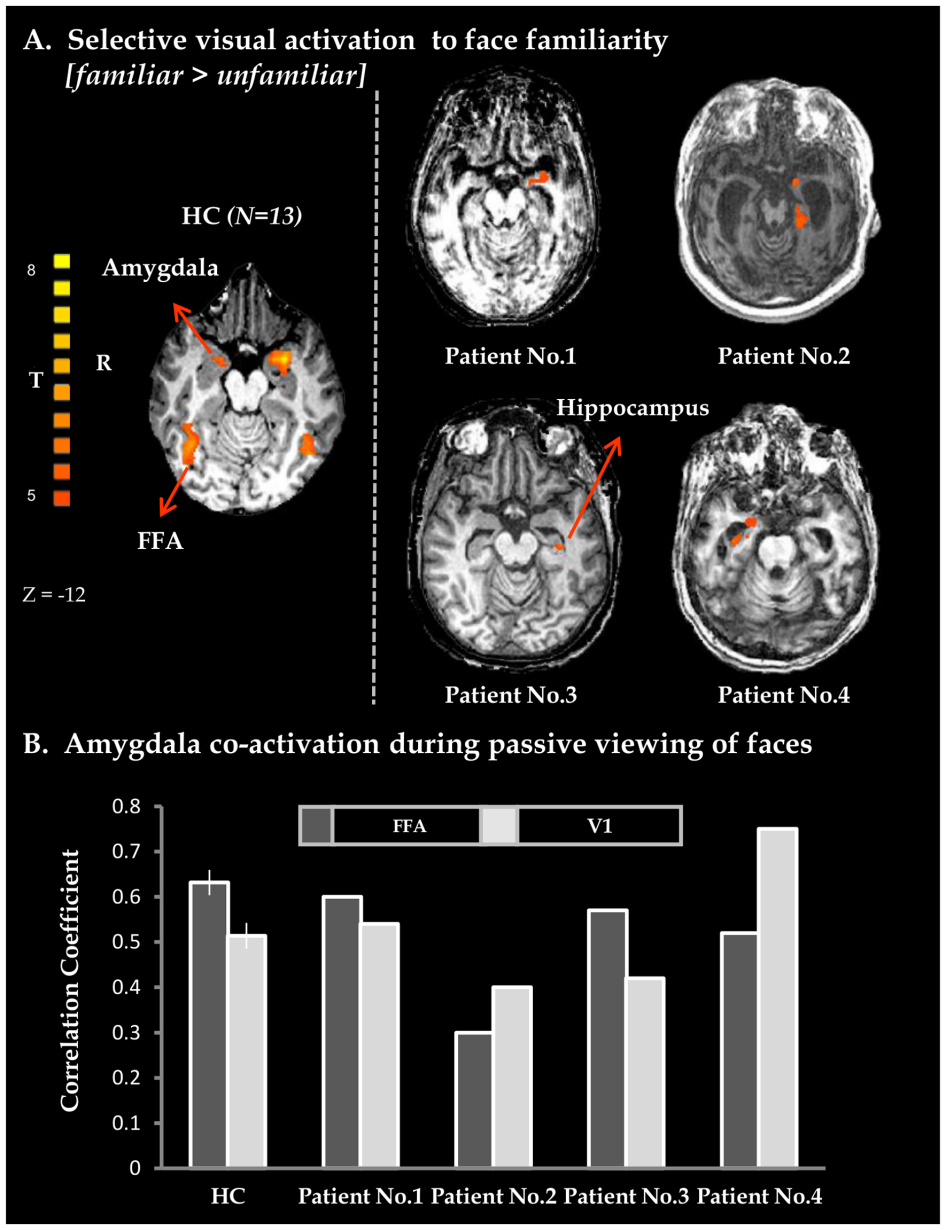
Emotional processing of familiar faces. A. Axial views of BOLD activation maps obtained from whole brain GLM analysis for Familiar vs. Unfamiliar Faces in 13 healthy controls (extreme left, random FDR corrected, shown at lower threshold) and for VS patients (patient 1–4, note that for patients 1 and 2 familiar faces included pictures of close others and self). Both in healthy controls and in VS patients limbic activations are evident. BOLD activity maps for patients were superimposed on each individual's structural image. B. Average correlation-coefficient measures representing limbic (amygdala) co-activation with FFA or with V1, obtained from whole brain functional connectivity analysis performed with seed activation derived from the amygdala. Vertical black line denote standard error in healthy controls (n = 13). Note in this study that limbic-FFA but not Limbic-V1 corresponds with patients' prognosis (see Table 1). HC – healthy controls; BOLD – blood-oxygenation-dependent level; FFA- fusiform face area; V1 – primary visual cortex.

**Figure 3:**
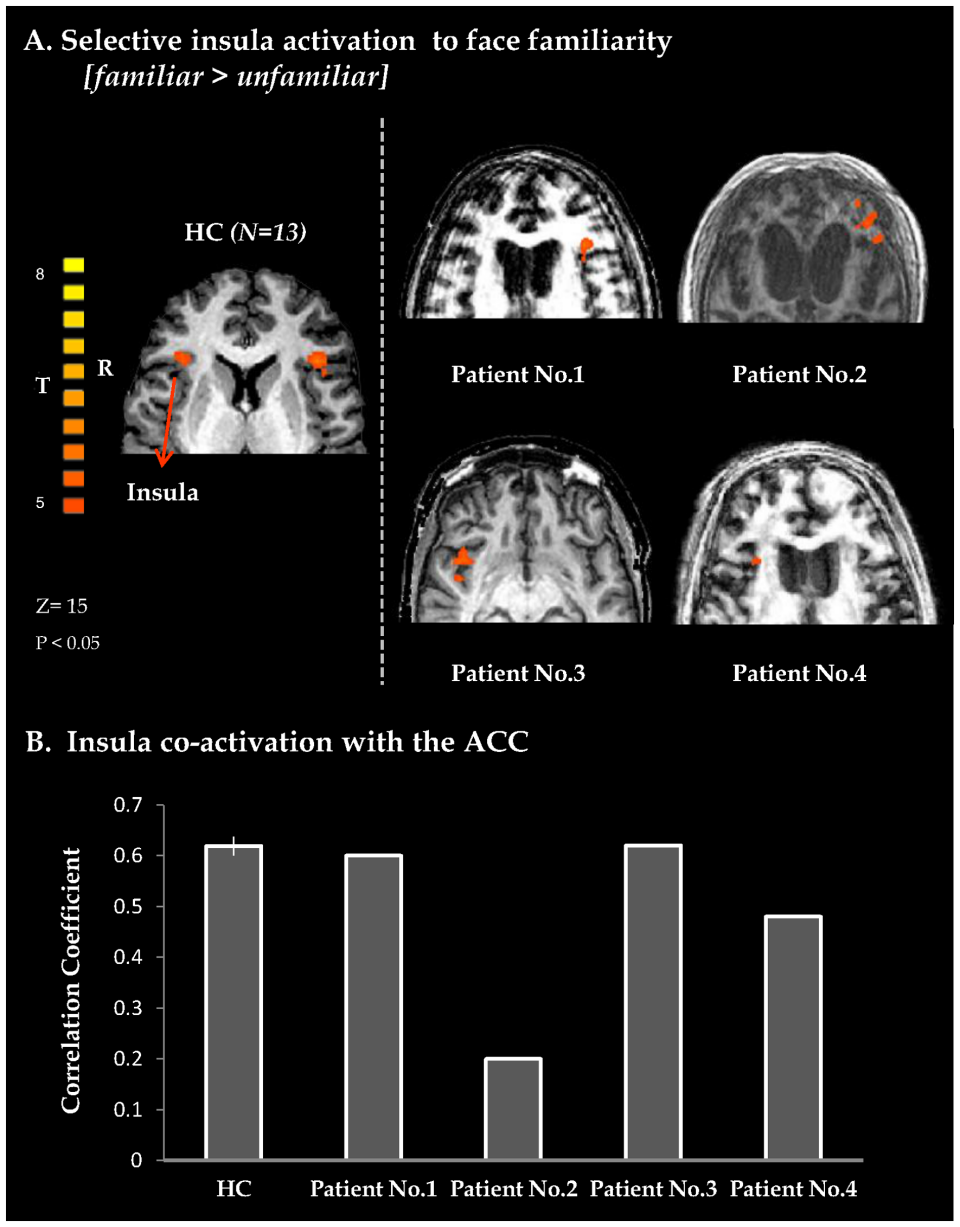
Self-aware processing of familiar faces. B. Axial views of insula BOLD activation obtained from whole brain GLM analysis with a contrast of familiar + self vs. unfamiliar faces, for a group of healthy controls (extreme left, random, FDR corrected (p<0.05), shown at lower threshold) and for individual VS patients. BOLD activity maps for patients were superimposed on each individual's structural image. B. Average correlation-coefficient measures for co-activation of the insula with ACC for HC and individual patients. Correlation coefficient values were obtained from whole brain functional connectivity analysis performed with seed activation derived from right anterior insula. Vertical black line in HC bar denotes standard error (n = 13). Note, that in this study, patients co-activation level corresponded with patients' prognosis (see Table 1). HC – healthy controls; BOLD – blood-oxygenation-dependent level; ACC – anterior cingulated cortex.

Additionally, healthy controls and all patients displayed widespread selective activations to face familiarity (see Figure 2, Figure 3 and Table S1) including limbic activity, mostly in the amygdala (Figure 2a) and cortical activity in the anterior insula (Figure 3a). Furthermore, in healthy controls as well as in patients, there was increased functional connectivity between the amygdala, V1 and the FFA (Figure 2B and table S2) compared to baseline, while the insula showed increased connectivity with the anterior cingulate cortex (ACC) (Figure 3b and table S2).

### Task 2– Familiar face Imagery

**Figure 4:**
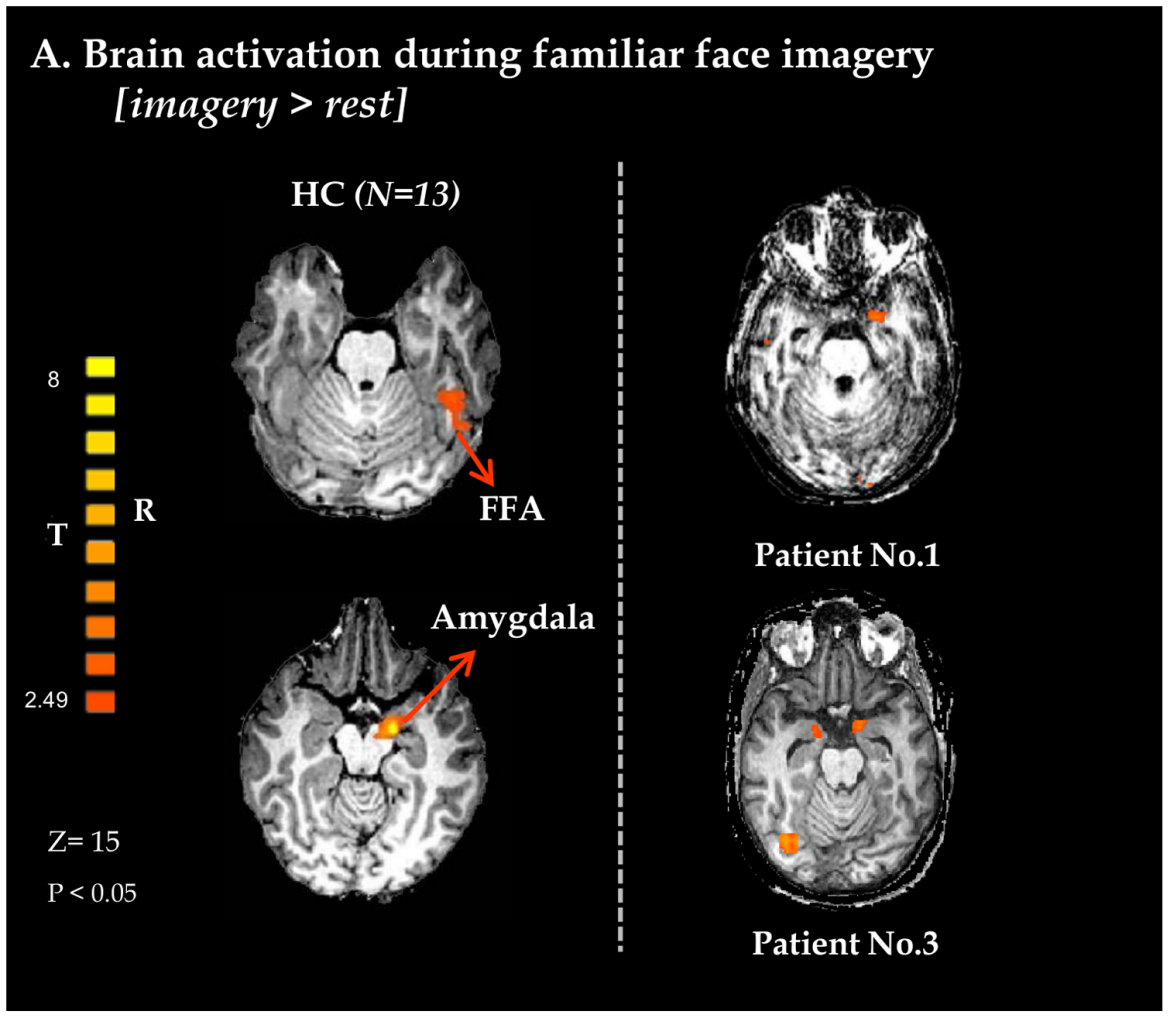
Emotional processing during imagery of familiar faces. Axial views of BOLD activation maps obtained from whole brain GLM analysis using a contrast of imagery vs. rest in 13 healthy controls (left views, random FDR corrected, shown at lower threshold) and two VS patients. In healthy controls and in patient 3 the maps present activation in both the FFA and amygdala, while in patient 1 only in the amygdala is activated at the same threshold. Bold activity maps for patients were superimposed on each individual's anatomical image. HC – healthy controls; FFA- fusiform face area; BOLD – blood-oxygenation-dependent level.

As expected, healthy controls exhibited activity in bilateral FFA, as well as bilateral amygdale, in response to familiar face imagery (see Figure 4). Two patients showed similar limbic activation during the guided imagery task. Of those, only patient 3 seemed to recruit both the FFA and amygdala, resulting in a map of activation which is indistinguishable from healthy controls. Patient 1, on the other hand, demonstrated robust activity in the left amygdala but without concomitant FFA activation.

## Discussion and Conclusions

The purpose of this study was to assess the possible existence of transient subjective mental states in VS patients by tracking emotional brain processing. To probe this possibility we used familiar and unfamiliar faces at two levels of task engagement: passive viewing and guided imagery. We expected that encoding face familiarity in both tasks would require high-order visual as well as cognitive and emotional processing. Our results reveal that VS patients, similarly to healthy controls, exhibit face specific activations in response to passively viewing faces, as well as limbic and salience activations in response to familiar face stimuli (specifically the amygdala and insula, respectively). Viewing familiar faces further elicited significant patterns of connectivity in both limbic and salience networks in patients as well as in healthy controls. In addition to these important activations, the active imagery task revealed top down modulation evident in two patients who exhibited limbic activations in response to personally familiar face imagery. Notably only these two patients later improved neurologically to a minimally conscious state.

Taken together, the selective brain activity and connectivity in emotional, perceptual and self related networks may signal the potential for large scale neural recruitment in some VS patients. Specifically, both the amygdala and the insula are considered major nodes in a salience brain network critical for cognitive control and attention modulation [16], [17], but also underlie unique processes in this regard. The amygdala is pivotal in processing emotional visual information, in particular decoding its significant value and meaning [17]. The insula, on the other hand, has been reported to be critical in recalling emotional memories [18] and in creating subjective awareness of emotional feeling, mostly in relation to its somatic aspects (i.e interoceptive awareness) [19]. These activations therefore might imply emotional monitoring of the environment, as well as the ability to access autobiographical memory.

### Passive viewing paradigm: activations recorded and the case for covert emotional awareness

Linking specific activations to subjective experiencing is still the subject of much debate [20] and therefore requires an examination of previous insights into the neural correlates of both visual and emotional awareness. Evidence that unseen stimuli undergo some unconscious processing in the primary and selective visual areas [21] as well as the amygdala [22] has previously been taken to imply that activity per se in these areas is not sufficient for conscious access. However, a recent study has shown that FFA activation to faces may in itself be a correlate of aware perception [23]. In the case of the amygdala as well, support for the notion that its activation is related to aware perception is growing [17]. Moreover, even if mere activity in these areas is insufficient, a differential increase in activity in the FFA and the amygdala, as was evident here for familiar faces, has been consistently linked to visual and emotional conscious attendance to emotional faces [24].

Familiar faces further elicited cortical activations in the anterior cingulate cortex and the anterior insula, previously implicated in high level sensory and emotional processing of familiar faces. High order activations to familiar faces are of crucial importance, as it has been widely proposed that access to consciousness requires top–down amplification from fronto-parietal areas to perceptual regions [25]. The ACC in particular has been frequently assumed as pivotal for aware perception and emotional regulation [26].

Furthermore, connectivity between the anterior insula and the ACC [10] as well as the amygdala and primary visual areas [27] have also been argued to be vital for emotional awareness. Specifically, co-activation of the anterior insula and the ACC has been proposed to compose a neural system vital for awareness of the self, as well as for processing emotions as part of face recognition [10].

Interestingly, the prognosis of patients in this study was reflected by the level of functional connectivity (Figure 2B, 3B) rather than by regional face selective activity. The small number of patients and their considerable clinical heterogeneity does not enable us to draw any strong conclusions regarding prognostic factors at this stage. Further studies should be conducted in order to ascertain the prognostic value of interregional connectivity in these patients. However, despite the fact that overall cortico-cortical connectivity cannot be accurately inferred from the connectivity analyses performed in this study, this finding does echo recent theoretical and empirical evidence that link different degrees of dysfunction in brain connectivity to the pathogenesis of different chronic disorders of consciousness, as well as improvement in connectivity to neurological recovery [1], [28]–[31]. These observations are compatible with the growing notion that the substrate of brain function may not be represented by regional activity but by activity within networks [17]. Apart from prognostic considerations, characterizing individual patients in terms of system-networking may prove clinically valuable considering the recent efforts to develop novel neurostimulation therapies in disorders of consciousness [29], [30] (e.g. in patient selection).

The fact that emotionally salient stimuli (e.g. personally familiar faces) elicited widespread brain responses while unfamiliar ones did not echoes the well known processing preference of emotional stimuli, which is a primal feature of brain function. Accordingly, it may indicate that some VS patients are indeed neurologically unresponsive for the most part but experience transient spells of awareness when presented with specific, emotionally salient, stimuli. This perhaps resembles a state of non-REM sleep in which short spells of awakenings occur, albeit with no behavioral signs attesting to the change in awareness. Such a suggestion is in line with the notion that behavioral-based diagnosis of VS groups together patients with differing degrees of neurological abilities, including some who are in fact more akin to a “non-responsive Minimally Conscious State (MCS)” [1], [5]. This may also better explain the clinical observation that improved VS patients almost always transition to MCS, characterized by exactly such intermittent and fluctuating level of responsiveness to certain stimuli rather than a continuous and consistent one [32].

### The active imagery task: voluntary modulation of brain activity

Although the specific activations exhibited in the passive viewing task are of great importance they cannot be considered as unequivocal evidence for covert emotional awareness. As consciousness is a first person experience, the possibility of making concrete statements about consciousness based on third person data is limited [11], [20]. This metaphysical gap is a theoretical limitation of all passive brain activation paradigms [11]. With this in mind, we also applied an active paradigm approach using the guided imagery task. During imagination of personally familiar faces all controls and one patient (patient 3) activated the FFA, indicating top down cognitive modulation elicited by the guided imagery. Furthermore, activations in the amygdala were evident in all healthy subjects as well as two patients (1 and 3), indicating emotional salience. Patient 1, who responded with only robust amygdala activation, may have possibly managed to recruit emotional processing but failed to fully operate cognitive-perceptual modulation (see Figure 3). These results suggest that emotional processing in VS patients can be driven willingly by internal mental processes. Specifically, the activations documented in patient 3 are of paramount importance. Based on prior reports discussing imagery tasks in VS patients, patient 3′s voluntary modulation of brain activity are truly indicative of covert awareness. Selective activation of emotional brain networks when voluntarily performing a complex cognitive task known to elicit emotion, provides compelling evidence that the cognitive content was accompanied by a subjective emotional experience. Such a finding may prove invaluable in the on-going treatment of VS patients and help guide rehabilitation or intervention efforts aimed to enhance moments of awareness in selected patients. Furthermore, apart from allowing us to possibly better patient care, it promises a deeper understanding of the nature of disorders of consciousness, and so a better understanding of the nature of consciousness in general.

## Supporting Information

Table S1 Healthy Control- BOLD Activation Coordinates.
(DOC)

Table S2 Healthy Controls- Functional Connectivity Results.
(DOC)
