# Supplementary material for: Emotional Processing of Personally Familiar Faces in the Vegetative State
Source: PLoS One. 2013 Sep 25;8(9):e74711. doi: 10.1371/journal.pone.0074711 (PMC3783455; doi:10.1371/journal.pone.0074711)
Supplement: Table S1 — Healthy Control- BOLD Activation Coordinates. (DOC) [file pone.0074711.s001.doc]

**Table S1.** Healthy Control- BOLD Activation Coordinates

| **Anatomical region\BA** | **Side** | ***T* value** | ***P* value** | **Talairach Coordinates** | | |
| --- | --- | --- | --- | --- | --- | --- |
|  |  |  |  |  |  |  |
| ***Faces Vs. Patterns*** |  |  |  | ***x*** | ***y*** | ***z*** |
| **Precuneus** | **R** | 8.165 | 0.000 | 5 | -62 | 18 |
| **Angular gyrus** | **R** | 8.761 | 0.000 | 47 | -59 | 15 |
| **Fusiform Face Area** | **R** | 6.790 | 0.000 | 35 | -56 | -15 |
| **Middle frontal gyrus** | **R** | 8.233 | 0.000 | 26 | 13 | 54 |
| **Parahippocampal gyrus** | **R** | 9.145 | 0.000 | 23 | -62 | -12 |
| **Postcentral gyrus** | **R** | -10.137 | 0.000 | 11 | -35 | 61 |
| **Precentral gyrus** | **R** | 7.231 | 0.000 | 32 | -2 | 30 |
| **Pulvinar** | **R** | 7.290 | 0.000 | 17 | -32 | 3 |
| **Sup. Frontal gyrus\ BA 8** | **R** | 6.068 | 0.000 | 11 | 34 | 45 |
| **Sup. Frontal gyrus (mPFC) \ BA9** | **R** | 9.132 | 0.000 | 8 | 46 | 33 |
| **Angular gyrus** | **L** | 5.223 | 0.000 | -40 | -71 | 15 |
| **Fusiform Face Area** | **L** | 8.237 | 0.000 | -37 | -59 | -12 |
| **Globus pallidus** | **L** | 6.797 | 0.000 | -10 | 1 | -6 |
| **Parahippocampal gyrus** | **L** | 6.308 | 0.000 | -25 | -62 | -15 |
| **Postcentral gyrus** | **L** | -7.828 | 0.000 | -19 | -29 | 61 |
| **Precentral gyrus** | **L** | 6.495 | 0.000 | -37 | -2 | 36 |
| **Pulvinar** | **L** | 7.299 | 0.000 | -22 | -35 | -3 |
| **Sup. Frontal gyrus \BA 6** | **L** | 6.246 | 0.000 | -7 | 10 | 45 |
| **Cuneus (V1)** | **L** | 12.379 | 0.000 | -7 | -89 | -12 |
| **Sup. Frontal gyrus (mPFC) \BA 10** | **L** | 7.309 | 0.000 | -7 | 58 | 6 |
| ***Familiar Vs. Unfamiliar faces*** |  |  |  |  |  |  |
| **Inferior Frontal gyrus** | **R** | 8.145 | 0.000 | 41 | 13 | 18 |
| **Fusiform Face Area** | **R** | 9.564 | 0.000 | 35 | -56 | -18 |
| **Sup. Frontal gyrus (mPFC )** | **R** | 10.216 | 0.000 | 5 | 46 | 24 |
| **Caudate nucleus** | **R** | 7.334 | 0.000 | 8 | 13 | 9 |
| **Hippocampus** | **R** | 7.579 | 0.000 | 20 | -35 | -6 |
| **Amygdala** | **R** | 7.907 | 0.000 | 11 | -5 | -9 |
| **Angular g** | **R** | 6.514 | 0.000 | 47 | -71 | 6 |
| **Precuneus** | **L** | 11.385 | 0.000 | -10 | -68 | 21 |
| **Sup. Frontal gyrus (mPFC ) \ BA 32** | **L** | 13.112 | 0.000 | -7 | 28 | 42 |
| **Sup. Frontal gyrus (mPFC ) \ BA 11** | **L** | 7.729 | 0.000 | -7 | 52 | 0 |
| **Sup. Frontal gyrus (mPFC ) \ BA 32** | **L** | 7.138 | 0.000 | -7 | 40 | 27 |
| **Hippocampus** | **L** | 9.457 | 0.000 | -13 | -26 | -9 |
| **Amygdala** | **L** | 9.245 | 0.000 | -19 | -5 | -6 |
| **Fusiform Face Area** | **L** | 11.253 | 0.000 | -40 | -56 | -18 |
| **Angular gyrus** | **L** | 9.169 | 0.000 | -40 | -65 | 21 |
| **Middle Temporal gyrus** | **L** | 8.731 | 0.000 | -55 | -11 | -12 |
| **Caudate nucleus** | **L** | 9.676 | 0.000 | -10 | 13 | 9 |
| **Middle frontal gyrus** | **L** | 8.540 | 0.000 | -37 | 17 | 33 |
| ***Face Imagination Vs. Rest*** |  |  |  |  |  |  |
| **Sup. Temporal gyrus (A1)** | **R** | 17.637 | 0.000 | 53 | -17 | 2 |
| **Angular gyrus** | **R** | -4.783 | 0.001 | 41 | -77 | 3 |
| **Cuneus** | **R** | -8.539 | 0.000 | 17 | -81 | 39 |
| **Anterior insula** | **R** | 3.849 | 0.003 | 35 | 18 | 12 |
| **Sup. Frontal gyrus \BA 8** | **R** | -6.376 | 0.000 | 17 | 16 | 52 |
| **Paracentral lobule** | **R** | -5.067 | 0.000 | 5 | -41 | 33 |
| **Sup. Frontal gyrus \BA 6** | **R** | 4.619 | 0.001 | 8 | 7 | 51 |
| **Precuneus** | **R** | -8.039 | 0.000 | 17 | -65 | 21 |
| **Thalamus** | **R** | 3.882 | 0.003 | 8 | -11 | 10 |
| **Sup. Frontal gyrus \BA 6** | **MID** | 5.192 | 0.000 | 1 | 1 | 60 |
| **Precuneus** | **L** | -4.432 | 0.001 | -19 | -62 | 18 |
| **Sup. Frontal gyrus \BA 6** | **L** | 7.591 | 0.000 | -6 | 10 | 51 |
| **Parahippocampal gyrus** | **L** | 4.584 | 0.001 | -28 | -23 | -27 |
| **Angular gyrus** | **L** | -5.912 | 0.000 | -28 | -83 | 3 |
| **Inferior Frontal gyrus** | **L** | 8.415 | 0.000 | -40 | 16 | 18 |
| **Sup. Temporal gyrus** | **L** | 12.303 | 0.000 | -37 | -32 | 6 |
| **Inferior Temporal gyrus** | **L** | 5.885 | 0.000 | -49 | -41 | -15 |
| **Fusiform Face Area** | **L** | 4.146 | 0.002 | -40 | -47 | -24 |
| **Thalamus** | **L** | 7.074 | 0.000 | -13 | -11 | 9 |
| **Amygdala** | **L** | 13.342 | 0.000 | -13 | -11 | -9 |
