# Supplementary material for: Emotional Processing of Personally Familiar Faces in the Vegetative State
Source: PLoS One. 2013 Sep 25;8(9):e74711. doi: 10.1371/journal.pone.0074711 (PMC3783455; doi:10.1371/journal.pone.0074711)
Supplement: Table S2 — Healthy Controls- Functional Connectivity Results. (DOC) [file pone.0074711.s002.doc]

**Table S2.** Healthy Controls- Functional Connectivity Results

| **Anatomical region\BA** | **Side** | ***T* value** | ***P* value** | **Talairach Coordinates** | | |
| --- | --- | --- | --- | --- | --- | --- |
|  |  |  |  |  |  |  |
| ***Right FFA Whole Brain Correlations*** |  |  |  | ***x*** | ***y*** | ***z*** |
| **Posterior middle Temporal gyrus** | **R** | 12.847 | 0.000 | 56 | -56 | 6 |
| **Lateral Precentral gyrus** | **R** | 17.391 | 0.000 | 47 | -2 | 33 |
| **Inferior Frontal gyrus** | **R** | 11.478 | 0.000 | 41 | 28 | 21 |
| **Sup. Occipital gyrus** | **R** | 11.211 | 0.000 | 14 | -92 | 3 |
| **Sup. Frontal gyrus (preSMA)** | **R** | 20.094 | 0.000 | 5 | 7 | 54 |
| **Putamen** | **R** | 11.130 | 0.000 | 20 | 1 | 3 |
| **Globus pallidus** | **R** | 14.254 | 0.000 | 11 | 1 | 11 |
| **Thalamus** | **R** | 15.137 | 0.000 | 11 | -14 | 9 |
| **Subthalamic nucleus** | **R** | 19.838 | 0.000 | 8 | -17 | -3 |
| **Hippocampaus** | **R** | 19.476 | 0.000 | 20 | -20 | -9 |
| **Amaygdala** | **R** | 11.111 | 0.000 | 17 | -10 | -15 |
| **Fusiform Face Area** | **R** | 16.212 | 0.000 | 32 | -47 | -18 |
| **Dorsal cingulate gyrus** | **MID** | 15.831 | 0.000 | -1 | 1 | 33 |
| **Precentral gyrus** | **L** | 13.605 | 0.000 | -43 | -11 | 54 |
| **Globus pallidus** | **L** | 14.635 | 0.000 | -19 | -2 | 9 |
| **Putamen** | **L** | 14.635 | 0.000 | -19 | -2 | 9 |
| **Thalamus** | **L** | 9.269 | 0.000 | -7 | -17 | 12 |
| **Subthalamic nucleus** | **L** | 17.181 | 0.000 | -7 | -23 | 0 |
| **Hippocampaus** | **L** | 17.924 | 0.000 | -28 | -20 | -6 |
| **Amaygdala** | **L** | 20.211 | 0.000 | -21 | -8 | -18 |
| **Fusiform Face Area** | **L** | 14.061 | 0.000 | -37 | -50 | -21 |
| ***Right Amygdala Whole Brain Correlations*** |  |  |  |  |  |  |
| **Middle temporal gyrus** | **R** | 11.586 | 0.000 | 44 | -14 | -15 |
| **Posterior cingulate gyrus (Isthmus)** | **R** | 15.022 | 0.000 | 8 | -47 | 3 |
| **Precuneus** | **R** | 11.365 | 0.000 | 5 | -59 | 15 |
| **Cuneus (V1)** | **R** | 13.821 | 0.000 | 11 | -77 | -9 |
| **Fusiform Face Area** | **R** | 15.595 | 0.000 | 38 | -56 | -21 |
| **Hippocampus** | **R** | 13.567 | 0.000 | 20 | -14 | -9 |
| **Amygdala** | **R** | 17.324 | 0.000 | 20 | -5 | -12 |
| **Middle Occipital gyrus** | **R** | 10.086 | 0.000 | 50 | -71 | 15 |
| **Middle Occipital gyrus** | **L** | 11.986 | 0.000 | -43 | -77 | 15 |
| **Cuneus (V1)** | **L** | 13.661 | 0.000 | -1 | -86 | -9 |
| **Fusiform Face Area** | **L** | 11.692 | 0.000 | -34 | -61 | -18 |
| **Hippocampus** | **L** | 13.669 | 0.000 | -28 | -20 | -9 |
| **Amygdala** | **L** | 12.631 | 0.000 | -25 | -5 | -12 |
| **Posterior cingulate gyrus (Isthmus)** | **L** | 13.474 | 0.000 | -10 | -47 | 6 |
| ***Left Insula Whole Brain Correlations*** |  |  |  |  |  |  |
| **Sup. Frontal gyrus (preSMA)** | **R** | 12.027 | 0.000 | 2 | 4 | 54 |
| **Precentral gyrus** | **R** | 13.092 | 0.000 | 38 | -2 | 45 |
| **Anterior Cingulate gyrus** | **R** | 12.050 | 0.000 | 5 | 19 | 31 |
| **Inferior Frontal gyrus** | **R** | 14.938 | 0.000 | 38 | 28 | 6 |
| **Anterior insula** | **R** | 11.651 | 0.000 | 29 | 7 | 6 |
| **Caudate nucleus** | **R** | 18.827 | 0.000 | 8 | 7 | 8 |
| **Thalamus** | **R** | 15.388 | 0.000 | 5 | -17 | 3 |
| **Pulvinar** | **R** | 19.707 | 0.000 | 17 | -30 | 6 |
| **Fusiform Face Area** | **R** | 13.588 | 0.000 | 29 | -59 | -23 |
| **Medial Temporal gyrus** | **R** | 17.757 | 0.000 | 53 | -65 | 3 |
| **Precentral gyrus** | **L** | 14.372 | 0.000 | -46 | -11 | 48 |
| **Anterior Cingulate gyrus** | **L** | 11.332 | 0.000 | -7 | 25 | 24 |
| **Anterior Insula** | **L** | 14.742 | 0.000 | -25 | 13 | -2 |
| **Caudate nucleus** | **L** | 14.401 | 0.000 | -10 | 4 | 6 |
| **Thalamus** | **L** | 17.145 | 0.000 | -10 | -17 | 0 |
| **Pulvinar** | **L** | 18.127 | 0.000 | -18 | -32 | -3 |
| **Fusiform Face Area** | **L** | 9.948 | 0.000 | -32 | -54 | -18 |
| **Inferior Frontal gyrus** | **L** | 13.691 | 0.000 | -37 | 19 | 6 |
